# The Baby’s First Week*Read before the Brazos Valley Medical Association, Hearne, November 14 and 15, 1899.

**Published:** 1900-01

**Authors:** H. W. Cummings

**Affiliations:** Hearne, Texas


For Texas Medical Journal.

### The Baby's First Week.*

BY H. W. CUMMINGS, M. D., HEARNE, TEXAS.

This short paper is intended only as an introductory to the important subject of the care and treatment of babies, which will be more elaborately discussed by the gentlemen who follow, in the continuance of the subject, taking up where I leave off and following it beyond the baby's second summer.

I merely desire to name a few points of interest touching the care of the baby during its first week, and make some suggestions, which, if more closely followed by us in our daily practice, will save much suffering by the little fellows, as well as prove of vast help to the mother. The first week is only the beginning of the responsible task of raising the baby.

I am a great believer in nature as a guide, and thus will advocate a very simple and practical course, yet strange to say, one which is observed by few parents, as well as some doctors, in the care of the newly born.

With the assurance of your patience, I will give rapidly the technique I usually observe at the baby's birth. The main object we must keep in mind is that our attention should be such as to develop a strong and healthy baby, so we will begin at birth, by insuring all the vitality possible. Do not be in too great haste to sever the cord, allowing the child to receive all the blood from the placenta possible with which to establish an independent life. When you are sure this object is attained, sever cord, and dress same with some dry antiseptic dressing, and place bandage sufficiently tight to retain dressing, and insure against hernia. The skin around the cord may be oiled so as to keep dressing from sticking to body.

A second point of importance in the child's development is a proper expansion of lungs and sufficient supply of oxygen. Nature in her wisdom makes the cry, the first effort of the baby, which usually insures this point, but often we are called upon to assist in this matter. Clean the mouth and throat of mucus which often prevents the child's free breathing and give sudden shock by the applications of hot water or even cold water sprinkled upon chest suddenly. Usually this will accomplish your purpose.

---

*Read before the Brazos Valley Medical Association, Hearne, November 14 and 15, 1899.

The third point I desire to mention is the proper protection of the skin. Coming as the newly born from a habitat of such regular temperature, as that of the mother's womb, is it surprising that we may expect ill effect from improper care of the child's surroundings who has been so suddenly expelled into so variable a clime? With great care should the child be bathed and its body oiled, using as little friction as possible, and then dressed in soft, light, yet warm, clothing. Flannel should be placed next to body, and the child placed where it will not be often disturbed.

The eyes and mouth should receive especial attention, that they be thoroughly cleansed. The failure in this is generally the cause of inflammation of these organs from the acid secretions of the vagina.

The neglect of the points here mentioned in bathing and dressing are more frequently than we think the cause of catarrhal inflammations, colic, etc., as well as irritation of skin, so common among children.

In closing my part of this subject I desire to mention only one more point, and, while it will more properly come up in the paper to follow me, in this subject, I cannot refrain from mentioning it.

Many times, no doubt, in your experience, that after you have turned your attention to the mother, some old lady, in a tone expressing her profound confidence in its wisdom will ask, "Doctor, what kind of tea do you give the baby?" Of all times in a physician's professional life, this, I think, gentlemen, is the most opportune to do good. I shall not mention the exceptions where artificial feeding becomes a necessity; but, treating the ordinary and majority cases, I want to ask why do our lady friends want to give anything?

Nature again has made ample provision for the baby's maintenance, and, in my opinion, art has proven utterly incapable to provide as good food, to say nothing of finding a better one. We all know that it is grossly wrong to give artificial food to an infant when the mother gives a normal quantity and quality of breast milk. Then let us be sufficiently diligent in the discharge of our duty to not only advise, but insist upon mothers and nurses following our directions in so important a subject. There is no greater field for work than in this, for strange to say a large per cent. of women persist in feeding babies.

I do not believe that the many ill conditions attributed to teething, etc., are justly so; but I do believe the origin of all bowel troubles under two years old are due to some foreign substance which enters the mouth. They may be influenced in their severity by heat, cold, teething, etc., but the primary infection comes through the mouth.

Now, gentlemen, I have told you nothing new, but if my few words should impress some of you to more carefully guard these points and more earnestly enlist in the war against the abominable practice of feeding babies, I shall have accomplished my purpose.

